# Comparison of Five TSH-Receptor Antibody Assays in Graves’ disease: *results from an observational pilot study*

**DOI:** 10.1186/s12902-019-0363-6

**Published:** 2019-04-25

**Authors:** Tristan Struja, Rebecca Jutzi, Noemi Imahorn, Marina Kaeslin, Fabienne Boesiger, Alexander Kutz, Esther Mundwiler, Andreas Huber, Marius Kraenzlin, Beat Mueller, Christian Meier, Luca Bernasconi, Philipp Schuetz

**Affiliations:** 1 Medical University Department, Clinic for Endocrinology, Diabetes & Metabolism, Kantonsspital Aarau, Tellstrasse, CH-5001 Aarau, Switzerland; 2 Department of Laboratory Medicine, Kantonsspital Aarau, Aarau, Switzerland; 3 Medical Faculty of the University of Basel, Basel, Switzerland; 4 Endonet, Basel, Switzerland

**Keywords:** Thyroid, Graves’ disease, TRAb, Bioassay

## Abstract

**Background:**

Early diagnosis and relapse prediction in Graves’ disease influences treatment. We assessed the abilities of four TSH-receptor antibody tests [TRAb] and one cyclic adenosine monophosphate bioassay to predict relapse of Graves’ disease.

**Methods:**

Observational study investigating patients presenting with Graves’ disease at a Swiss hospital endocrine referral center or an endocrine outpatient clinic. Main outcomes were diagnosis and relapse of Graves’ disease after stop of anti-thyroid drugs. We used Cox regression to study associations of TRAb levels with relapse risk and calculated c-statistics [AUC] to assess discrimination. Blood draws took place as close as possible to treatment initiation.

**Results:**

AUCs ranged from 0.90 (TSAb Biossay by RSR) to 0.97 (IMMULITE TSI by Siemens). Highest sensitivity (94.0%) was observed for IMMULITE TSI and RSR TRAb Fast, while the greatest specificity (97.9%) was found with the EliA anti-TSH-R (by Thermo Fisher). In Cox regression analysis comparing the highest versus the lower quartiles, the highest hazard ratio [HR] for relapse was found for BRAHMS TRAK (by Thermo Fisher) (2.98, 95% CI 1.13–7.84), IMMULITE TSI (2.40, 95% CI 0.91–6.35), EliA anti-TSH-R (2.05, 95% CI 0.82–5.10), RSR Fast TRAb (1.80, 95% CI 0.73–4.43), followed by RSR STIMULATION (1.18, 95% CI 0.46–2.99). Discrimination analyses showed respective AUCs of 0.68, 0.65, 0.64, 0.64, and 0.59.

**Conclusion:**

The assays tested had good diagnostic power and relapse risk prediction with few differences among the new assays. Due to the small sample size and retrospective design with possible selection bias, our data need prospective validation.

**Electronic supplementary material:**

The online version of this article (10.1186/s12902-019-0363-6) contains supplementary material, which is available to authorized users.

## Introduction

Graves’ disease [GD] is among the leading causes of hyperthyroidism affecting approximately 0.5% of the general population, especially young women [1]. It is caused by the presence of autoantibodies to the thyrotropin [TSH] receptor leading to unregulated production and secretion of thyroid hormones [1]. Typically, GD is characterized by suppressed serum TSH and overproduction of thyroid hormones thyroxine and triiodothyronine [T4 and T3] [2]. To distinguish GD from other causes of hyperthyroidism, measurement of TSH-receptor autoantibodies [TRAb] is usually helpful [3].

Most patients are initially treated with antithyroid drugs [ATD] for a recommended duration of 12 to 18 months, but this therapy may have adverse effects such as agranulocytosis, rash, joint pain, and hepatitis [4]. Other treatment options like radioactive iodine or total thyroidectomy are preferred in patients with relapse after ATD, however, these treatments usually lead to persisting hypothyroidism and lifelong T4-replacement [1, 2]. Importantly, the rate of relapse after ATD is high (around 50%) [1]. To predict relapse in GD, the Graves’ Recurrent Events After Therapy [GREAT] score has been proposed and recently validated by our research group [5, 6]. This score is based on clinical and biochemical parameters. Age at diagnosis (≥40 years), higher serum fT4 (≥40 pmol/L), higher serum TRAb (≥6 U/L), and larger goiter sizes (WHO class II–III) were associated with higher recurrence rates. In the original study, the GREAT score discriminated patients with relapse from those without relapse with a fair prognostic accuracy area under the curve [AUC] of 0.67 (95% confidence interval [CI]: 0.54–0.77). There was a 68% risk of relapse in patients with class III (4–6 points in the GREAT score) compared to 16% in patients with class I (0–1 points in the GREAT score) and 44% in patients with class II (2–3 points in the GREAT score) [5]. In addition to this clinical score, pooled evidence from a systematic review and meta-analysis showed that elevated first to third generation assay TRAb levels at diagnosis are associated with higher relapse rates [7].

Recently, two new fully automated TRAb immunoassays have become available, IMMULITE TSI (Siemens Healthineers) and EliA anti-TSH-R (Thermo Fisher Scientific). The former uses recombinant thyrotropin receptor chimeras and is based on a bridge technology. The latter is based on immunological competitive reactions between patient’s autoantibodies and human monoclonal antibodies for the binding to human recombinant TSH receptors, similarly to BRAHMS TRAK and RSR Fast TRAb. Their sensitivity and specificity in the diagnosis of GD have been described to be high and comparable to other 3rd generation TRAb tests [8, 9]. The advantage of these new immunoassays is not only their automated routine, but in particular for the IMMULITE TSI its declared ability to specifically detect only TSH-receptor stimulatory antibodies. This property has been so far reserved to laborious bioassays [8, 10]. Yet, the utility of these new third-generation immunoassays in predicting GD relapse at diagnosis has not been assessed so far. Herein, we compared five different TSH-receptor antibody tests for their ability to diagnose and predict relapse of Graves’ disease.

## Methods

In this 10-year retrospective, observational cohort study we analyzed data from 332 patients from a large endocrine outpatient clinic and one hospital based endocrine tertiary referral center in Switzerland. The primary outcome of this study was relapse in GD after an ATD treatment cycle similar to a previous study [6]. Patients were treated with ATD in a titration regimen upon their first episode of hyperthyroidism. Inclusion criteria were a first episode of GD defined as suppressed serum TSH (< 0.01 mU/l), elevated fT4, and if available, diffuse increased uptake in thyroid scintigraphy (99 m-Tc-pertechnetate). Patients with follow-up after start of ATD treatment < 24 months, ATD treatment < 12 months, initial ablative therapy (i.e. surgery or RAI), and time gap between initiation of treatment and blood draw over 2.5 months were excluded. This left 83 GD patients for analysis. In the diseased control group, we included 48 patients with Hashimoto’s thyroiditis (*n* = 16), thyroid autonomy (*n* = 13), thyroiditis (*n* = 9), and other hyperthyroid-associated diseases (*n* = 10, i.e. toxic goiter, amiodarone-induced thyroiditis). Relapse had to be established by suppressed TSH and elevated peripheral hormone (fT4).

### Clinical data

We collected clinical data by medical charts and electronic records review and if necessary, we complemented missing follow-up data by phone calls to patients and general practitioners. We gathered the following clinical parameters from the first patient’s visit: goiter size (WHO classification, 0-III); thyroid volume assessed by sonography; date of first ATD and the type of drug used; smoking status (yes or no); presence of Graves’ orbitopathy (yes or no); anti-thyroperoxidase-antibodies [TPO-Ab]; TRAb levels; and whether any other autoimmune diseases were present. During the course of disease, we observed TSH levels in constant intervals, date of ATD withdrawal, changes in drug regimen and reasons therefore (i.e. adverse effects), date of relapse, and, if no relapse occurred, date of last consultation. All patients were usually treated for 12 to 18 months with carbimazole or propylthyouracil using a titration regime.

### Laboratory measurements

After blood withdrawal, samples were directly centrifuged and analyzed for serum TSH, fT4, anti-TPO-Ab, and TRAb levels by commercially available laboratory assays as part of the clinical routine in the different participating sites. Routine TRAb were either measured at the Kantonsspital Aarau or at Hormony (specialized laboratory on hormone analysis, Prof. J. Girard, Basel, Switzerland). The TRAb assays routinely used and their technical specifications are listed in Additional file 1: Table S1.

Leftover serum aliquots were stored at − 20° Celsius and mean duration storage time was 46 months (median 46 months; 17 to 70 months interquartile range). TRAb concentration was measured with the following assays according to the manufacturers’ instructions: BRAHMS TRAK human KRYPTOR (Thermo Fisher Scientific, Germany), IMMULITE 2000 TSI (Siemens, Healthineers, Germany), EliA anti-TSH-R (Thermo Fisher Scientific, Germany), and ELISA RSR TRAb Fast (RSR Limited, UK). Cut-offs suggested by the manufacturers were used to evaluate diagnostic performance. For the detection of stimulating type (TSAb) and blocking type (TSBAb) autoantibodies patient sera were shipped on dry ice to RSR Limited (UK) who performed CHO-cell based, cAMP-dependent bioassays with all samples in triplicates (BioassayRSR™ TSAb and TSBAb). Intracellular cAMP was subsequently determined using the Direct Cyclic AMP ELISA (Enzo Life Sciences, Switzerland). A stimulation of ≥150% compared to the healthy blood donor control was considered as a positive result for a stimulating activity. Blocking activity was present if ≥30% inhibition of TSH stimulation compared to the healthy blood donor control was observed. For more information on the assays tested, please refer to the appropriate section of the Additional file 1.

### Statistical analysis

We recently externally validated the GREAT score. Primary objective of our study was to compare the capability of the different TRAb assays in diagnosing GD and to analyze whether the GREAT score could be further improved by the addition of novel and more specific TRAb assays. For this purpose, we performed similar statistical analyses as described before [5]. In brief, we used univariate and multivariate Cox-proportional hazard regression models to study the association of previously suggested risk factors separately and combined in the GREAT score with the risk for time to relapse. For dichotomization of variables, we used the same cut-offs as in the original report, except for the new assays where separated data into four quantiles. We also calculated the GREAT score risk classes as suggested [5]. Kaplan-Meier method was used to graphically display data with use of the log-rank test. Area under the receiver operator curve [AUC] [ROC] over the whole follow up time after ATD stop was used to assess discriminative power of the GREAT score.

All significance tests were two-sided and *P* <  0.05 was considered statistically significant. Categorical variables are expressed as percentages (counts) and continuous variables as mean and standard deviation. If applicable, a 95% CI is provided. As our not normally distributed data was right-skewed, we log transformed (base 10) it before analysis. Survival analysis and ROC curves for relapse were conducted using Stata software version 12.1 (Stata Corp., College Station, TX, USA). Diagnostic performance of the different TRAb assays was analyzed using MedCalc Statistical Software version 15.11.4 (MedCalc Software bvba, Ostend, Belgium; https://www.medcalc.org; 2015).

## Results

### Baseline characteristics

We included 131 patients in this cohort (14.5% males). Out of the GD subpopulation, 18 (21.7%) had a relapse after a median follow-up time of 22 (9; 33 interquartile range [IQR]) months after ATD start, or 17 (7; 32 IQR) months after ATD withdrawal, respectively. To illustrate patient inclusion, we integrated a selection flow sheet into the appendix (see Additional file 1: Figure S1). Tables 1 and 2 shows details of the patient population stratified by relapse and diagnosis. Patients with relapse had a higher rate of endocrine orbitopathy, higher fT_4_, TPO-Ab, and TRAb levels. After relapse, all but two patients (they opted for surgery) chose to continue ATD treatment.

**Table 1:** Baseline characteristics of GD patients

Numbers (%)	GD no relapse	GD relapse	*P* - value^§^
Sex	65 (49.6%)	18 (13.7%)	
Female	55 (85%)	15 (83%)	0.89
Male	10 (15%)	3 (17%)	
Age (years), mean ± SD	52 ± 13	47 ± 13	0.13
BMI (kg/m^2^), mean ± SD	24 ± 4.6	25 ± 3.7	0.23
Smokers	9 (25%)	1 (20%)	0.81
Treatment duration (months), median (IQR)	19 (18, 21)	18 (17, 21)	0.81
Follow up duration after ATD stop (months), median (IQR)	11 (3, 36)	1 (0.5, 11)	< 0.01
Thyroid volume (ml), median (IQR)	14 (11, 18)	14 (9.6, 16)	0.71
Goiter size (WHO grade, 0-III)			
0	33 (62%)	10 (67%)	0.84
I	12 (23%)	4 (27%)	
II	7 (13%)	1 (7%)	
III	1 (2%)	0 (0%)	
Endocrine orbitopathy (N/%)	18 (28%)	7 (39%)	0.36
fT4 (pM), median (IQR)	30 (21, 36)	35 (20, 55)	0.31
T3 (pM), median (IQR)	3.5 (2.5, 4.4)	2.9 (2.5, 6.4)	0.90
fT3 (pM), median (IQR)	10 (7.7, 17)	21 (14, 29)	0.06
TPO-Ab (U/l), median (IQR)	89 (49, 475)	120 (90, 357)	0.49
Routine TRAb assay^a^ (U/L), median (IQR)	5.4 (2.8, 10)	12 (3.5, 27)	0.10
IMMULITE TSI (Cut-off 0.55 U/L), median (IQR)	3.4 (1.6, 7.5)	5.6 (3.6, 17)	0.04
BRAHMS TRAK (Cut-off 1.8 U/L), median (IQR)	4.6 (2.6, 11)	8.6 (5.1, 20)	0.02
EliA anti-TSH-R (Cut-off 2.9 U/L), median (IQR)	4.4 (2.9, 9.6)	7.4 (4.4, 13)	0.04
RSR TRAb Fast (Cut-off 1.0 U/L), median (IQR)	4.3 (2.7, 7.5)	6.9 (4.1, 16)	0.06
RSR-bioassay STIMULATION (Cut-off 150%), median (IQR)	461 (192, 835)	536 (291, 1419)	0.26
Additional autoimmune disease:			0.51
GIT (IBD, CD, pernicious anemia)	1	1	
T1DM	1	0	
Other	1	0	

^a^Originally, study centers used different commercially available assays with different cut-offs, for details please see Additional file 1**:** Table S1

^§^categorical and binary variables were compared by Pearson’s chi-squared test, continuous, non-normally distributed variables were compared by Wilcoxon rank-sum test; *P*-values not adjusted to multiple testing

**Table 2:** Baseline characteristics of the non-GD patients

Numbers (%)	Hashimoto’s Thyroiditis	Thyroiditis	Toxic nodular goiter	Other^a^	*P*-value^§^
Sex	16 (12.2%)	9 (6.9%)	13 (9.9%)	10 (7.6%)	
Female	16 (100.0%)	8 (89%)	12 (92%)	6 (60.0%)	0.23
Male	0 (0.0%)	1 (1%)	1 (8.0%)	4 (40.0%)	
Age (years), mean ± SD	52 ± 13	51 ± 15	68 ± 16	53 ± 22	0.04
BMI (kg/m^2^), mean ± SD	25 ± 3.7	25 ± 3.2	24 ± 3.3	23 ± 3.7	0.68
IMMULITE TSI (Cut-off 0.55 U/L), median (IQR)	0.05 (0.05, 0.09)	0.05 (0.05, 0.2)	0.05 (0.05, 0.05)	0.05 (0.05, 0.05)	0.20
BRAHMS TRAK (Cut-off 1.8 U/L), median (IQR)	0.2 (0.2, 0.2)	0.2 (0.2, 0.2)	0.2 (0.2, 0.62)	0.2 (0.2, 0.2)	0.88
EliA anti-TSH-R (Cut-off 2.9 U/L), median (IQR)	1.4 (1.4, 1.4)	1.4 (.9, 1.9)	1.4 (.9, 1.4)	1.4 (1.2, 1.7)	0.60
RSR TRAb Fast (Cut-off 1.0 U/L), median (IQR)	0.9 (0.9, 0.9)	0.9 (0.9, 1.1)	0.9 (0.9, 0.9)	0.9 (0.9, 1.1)	0.09
RSR-bioassay STIMULATION (Cut-off 150%), median (IQR)	94 (85, 119)	98 (91, 131)	87 (86, 92)	98 (93, 182)	0.12

Abbreviation: *CD* celiac disease, *GIT* gastrointestinal tract, *IBD* inflammatory bowel disease, *IQR* interquartile range, *pM* pmol/L, *SD* standard deviation, *T1DM* type 1 diabetes mellitus

^a^Other includes: amiodarone induced hyperthyroidism, euthyroid sick syndrome, postpartum thyroiditis, silent thyroiditis, euthyroid goiter, follicular and papillary carcinoma, functional TSH suppression after i.v. contrast agent

^§^categorical and binary variables were compared by Pearson’s chi-squared test, continuous, non-normally distributed variables were compared by Wilcoxon rank-sum test; *P*-values not adjusted to multiple testing

A total of 7.3% of the patients were initially treated with propylthyouracil, whereas the remainder received carbimazole. Ten patients were switched from carbimazole to propylthyouracil or vice versa. Most changes occurred because of pregnancies (*n* = 1) or skin rashes (*n* = 7). Besides one case of hepatitis (carbimazole group), no serious adverse effects occurred. Especially, there was no case of liver failure, agranulocytosis or death.

### Diagnostic performance

**Fig. 1:**
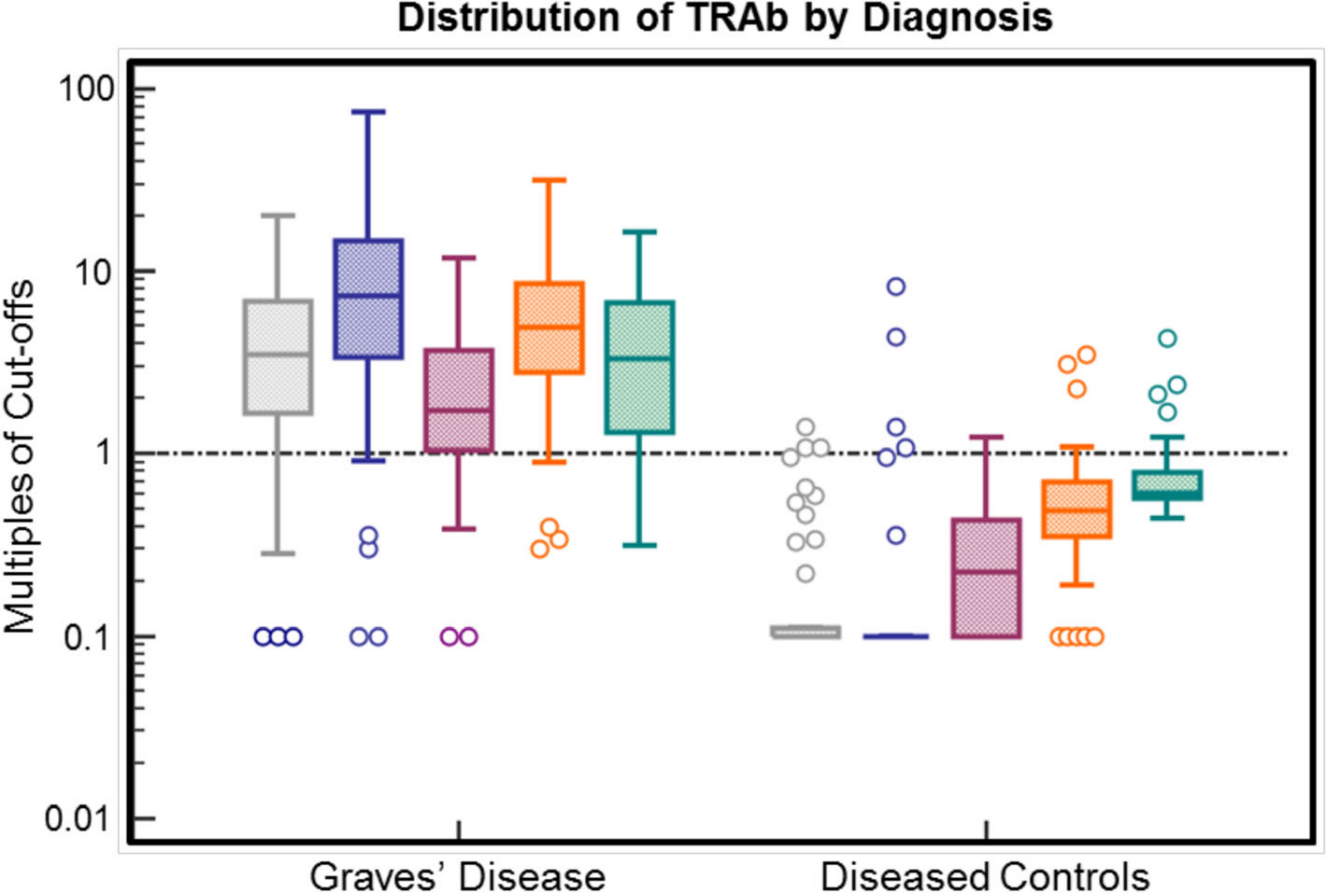
Distribution of TRAb levels in GD patients vs. diseased controls y-axis is on a logarithmic scale

**Fig. 2:**
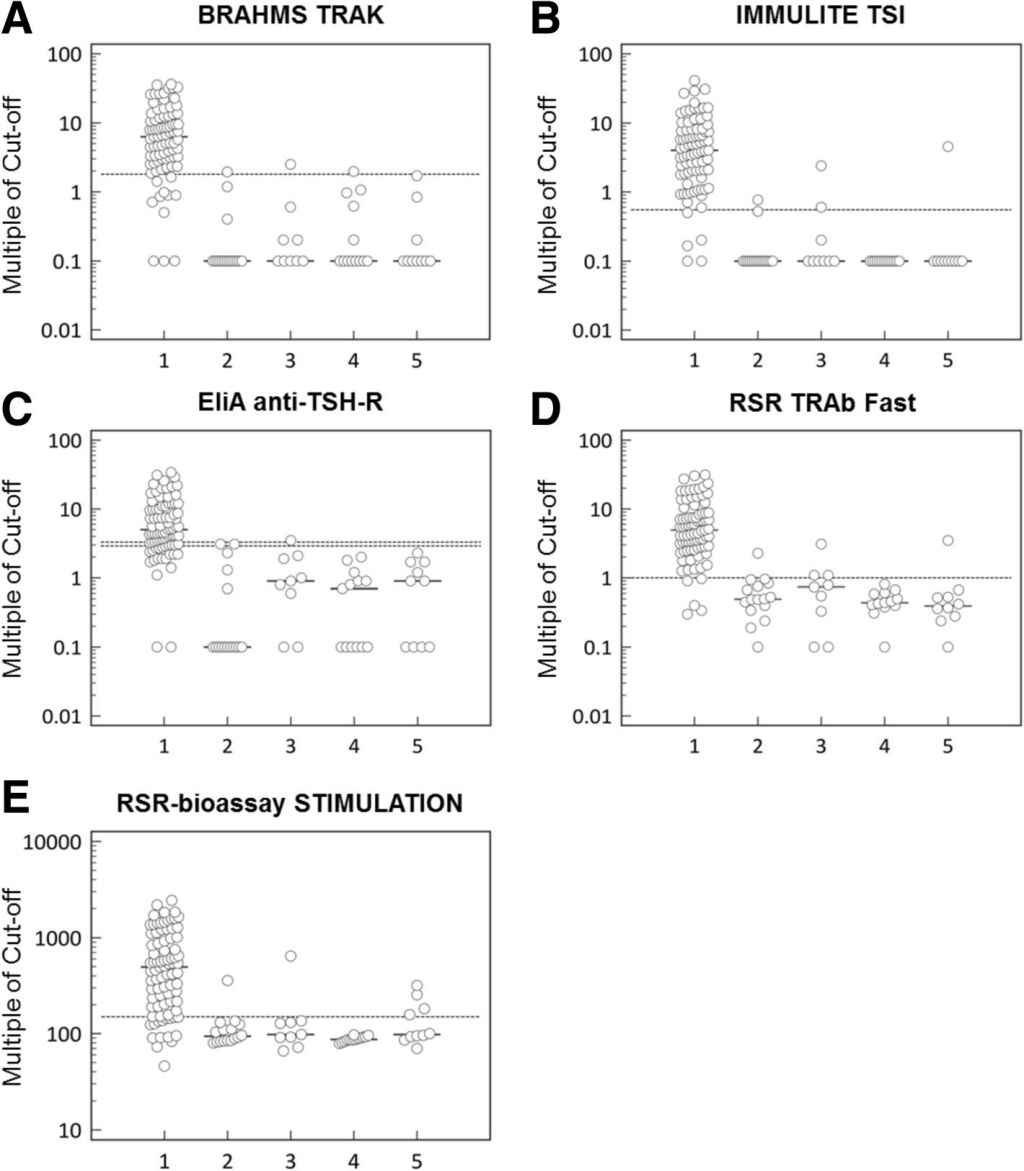
Distribution of TRAb levels by diagnosis y-axes are on a logarithmic scale. 1, Graves’ disease. 2, Hashimoto’s thyroiditis. 3, Thyroiditis. 4, Toxic nodular goiter. 5, Other (i.e. amiodarone induced hyperthyroidism, euthyroid sick syndrome, postpartum thyroiditis,silent thyroiditis, euthyroid goiter, follicular and papillary carcinoma, functional TSH suppression after i.v. contrast agent). Panel **a** TRAb from Brahms. Panel **b** TRAb from Siemens. Panel **c** TRAb from Thermo Fisher Scientific. Panel **d** TRAb from RSR Limited. Panel **e** TSAb from RSR Limited

The distribution of TRAb levels of the 83 GD patients and 48 diseased controls measured by the different assays is depicted in Fig. 1. ROC curve analysis revealed AUCs ranging from 0.90 (TSAb Biossay – RSR Limited) to 0.97 (IMMULITE TSI – Siemens) (Table 3). Highest sensitivity (94.0%) was observed for IMMULITE TSI (Siemens) and RSR TRAb Fast (RSR Limited) assays while the greatest specificity (97.9%) was found with the EliA anti-TSH-R (Thermo Fisher Scientific). Figure 2 shows the distribution of TRAb concentrations by diagnosis.

**Table 3:** AUC for GD diagnosis and relapse compared to GREAT score and refitted with new TRAb’s^b^

	DIAGNOSIS				GD RELAPSE PREDICTION			
Discriminator	Sensitivity [%]	Specificity [%]	AUC	95% CI	AUC	95% CI	Improved AUC	95% CI
GREAT score without routine TRAb					0.57	(0.43–0.71)		
GREAT score with routine TRAb^a^					0.69	(0.56–0.81)		
							GREAT score with new assay	
BRAHMS TRAK	86.7	93.7	0.96	(0.91–0.99)	0.71	(0.57–0.86)	0.67	(0.53–0.81)
IMMULITE TSI	94.0	91.7	0.97	(0.92–0.99)	0.69	(0.54–0.84)	0.66	(0.53–0.79)
EliA anti-TSH-R								
≥ 2.9 U/L	79.5	93.7	0.95	(0.90–0.98)	0.68	(0.52–0.83)	0.68	(0.54–0.82)
> 3.3 U/L	71.1	97.9	0.95	(0.90–0.98)				
RSR TRAb Fast	94.0	89.6	0.96	(0.91–0.99)	0.67	(0.50–0.83)	0.64	(0.50–0.78)
RSR-bioassay STIMULATION	81.9	87.5	0.90	(0.84–0.95)	0.62	(0.45–0.78)	0.62	(0.48–0.76)

Abbreviation: *GREAT* Graves’ Recurrent Events After Therapy, *ROC AUC* receiver operator curve, analysis under the curve, *TRAb* TSH-receptor autoantibodies

^a^Recalculated for this cohort

^b^ROC AUC with 95% CI < 50% are regarded as worse than chance; 50–70% are regarded as clinically unsuitable; > 70% are deemed clinically relevant

### Discrimination statistics for relapse assessment

**Fig. 3:**
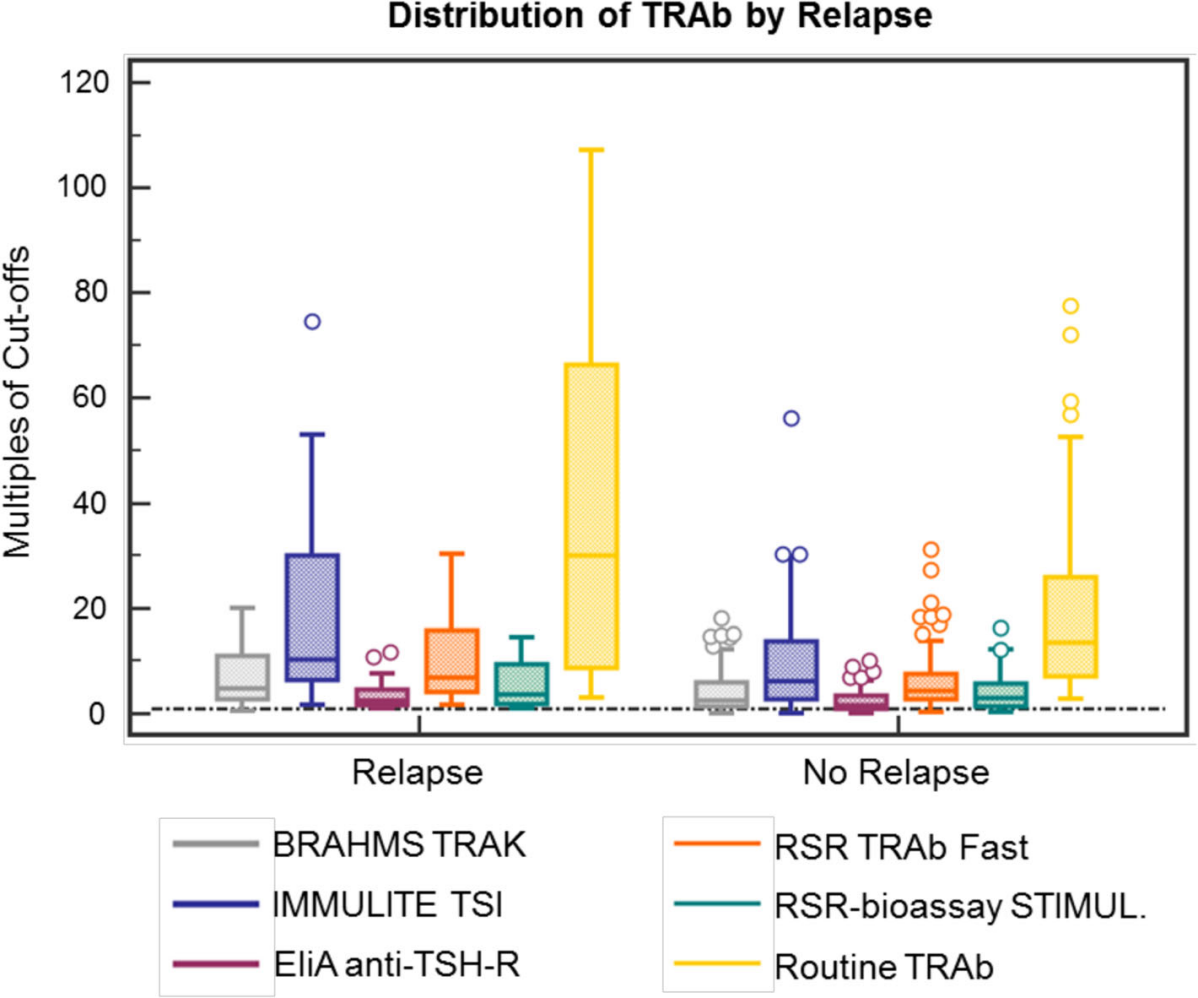
Distribution of TRAb levels at diagnosis according to relapse status. Median and IQR values according to the figure are presented in the first two columns of Table 1

Figure 3 shows distribution of TRAb levels of the 83 GD patients depicted. Median and IQR values according to the figure are presented in the first two columns of Tables 1 and 2. We calculated the AUCs to assess discrimination of assays in regard to prediction of relapse (see Additional file 1: Figure S2). AUC figures for the GREAT score were recalculated for our present cohort according to our initial publication (see Table 3) [6]. Most assays predicted the outcome relapse with moderate AUCs of around 0.67 to 0.71. Combined with the GREAT score, they did not show a significantly improved predictive ability. All assays performed in a similar range except for the bioassay.

### Cox proportional hazard regression analysis

**Fig. 4:**
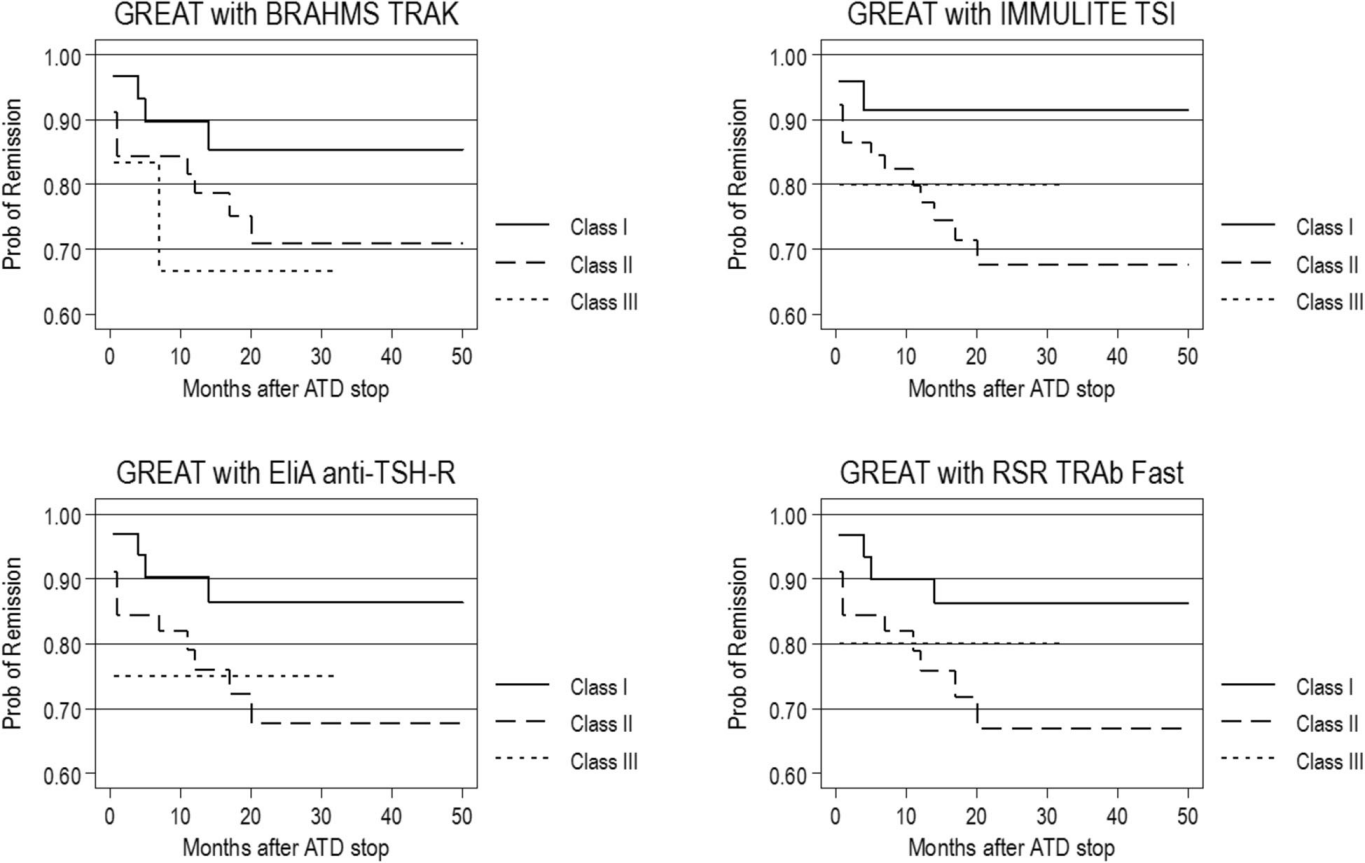
Kaplan-Meier-Survival graphs of GREAT score with new TRAb assay instead of routine assay

To analyze whether the TRAb assays further improve the predictive ability of the GREAT score, we modeled a univariate and a multivariate cox regression analysis. The results from the TRAb assays were split according to their quartiles and we compared the highest versus the remaining three quartiles (see Table 4). In univariate analysis, we modeled the TRAb level against time to relapse after ATD withdrawal. All assays showed significant associations but with very wide CI due to the small sample size. Incorporation of the TRAb assay results into a multivariate model (i.e. the existing GREAT score without the routine TRAb) provided improved hazard ratios with the BRAHMS assay as compared to the GREAT score with the routine TRAb. Whereas IMMULITE, EliA anti-TSH-R, and RSR TRAb Fast only improved the GREAT score for GREAT class II, but not class III. To illustrate these findings, we plotted Kaplan-Meier survival curves (see Fig. 4 and Additional file 1: Figure S3). Also, we further added either smoking or orbitopathy as covariates into the model. There were no significant changes in HRs (results not shown).

**Table 4:** Hazard ratios for relapse fitted with new TRAb’s 4th versus 1st-3rd quartile and into GREAT^a^

Assay	Recommended cut-offs by the manufacturer	Level of Q_4_	HR for assay alone (Q_4_ vs. Q_1–3_) (95% CI)	HR for GREAT Class II with new assay (95% CI)	HR for GREAT Class III with new assay (95% CI)
BRAHMS TRAK	≥ 1.80 U/L	≥ 8.10 U/L	3.53 (1.35–9.22)	2.02 (0.64–6.36)	3.11 (0.57–17.07)
IMMULITE TSI	≥ 0.55 U/L	≥ 5.66 U/L	3.12 (1.20–8.12)	3.73 (0.84–16.44)	3.01 (0.27–33.34)
EliA anti-TSH-R	≥ 2.90 U/L	≥ 7.40 U/L	4.52 (1.71–11.99)	2.44 (0.79–7.60)	2.37 (0.26–21.18)
RSR TRAb Fast	≥ 1.00 U/L	≥ 7.21 U/L	4.41 (1.66–11.71)	2.47 (0.80–7.72)	1.96 (0.22–17.62)
RSR-bioassay STIMULATION	≥ 150%	≥ 711%	3.63 (1.39–9.46)	N/A	N/A

^a^Class I serves as reference

## Discussion

Based on this observational, secondary analysis of blood samples collected in a representative patient population from a multicenter study, we tested five TRAb assays for their power to diagnose and predict relapse in GD patients. Three competition assays, including the recently released automated EliA anti-TSH-R, an automated assay based on bridge technology [9], and one cell-based bioassay have been considered.

### Diagnosis of GD

ROC curve analysis demonstrated highly comparable AUCs for the different assays except for the bioassay which showed a fairly lower AUC. Sensitivities varied from 79.5% (EliA anti-TSH-R) to 94.0% (IMMULITE TSI and RSR TRAb Fast). Previous studies described slightly higher sensitivities for IMMULITE TSI between 95 and 100% [8, 10–12], while the manufacturer suggested a sensitivity for EliA anti-TSH-R varying between 83% at a cut-off 2.9 U/l and 79% at 3.3 U/l (grey-zone 2.9–3.3 U/l) [13]. Thus, in our study, the performance of the bioassay was inferior to that reported in former studies examining different bioassay systems [12, 14, 15]. BRAHMS TRAK showed a higher sensitivity than previously reported by Diana et al. [12]. The RSR TRAb Fast, a modified version of the RSR 3rd generation TRAb ELISA [16], exhibited a sensitivity of 94% which is higher than the 85–93% observed with the unmodified assay [17, 18]. Overall, we report lower sensitivities compared to those described in a meta-analysis performed by Tozzoli et al. [19] examining different 3rd generation assays (pooled sensitivity of 97.4%). There are several explanations for these differences. First, we evaluated a rather small cohort of patients and due to the retrospective design, selection bias towards lower severity patients is likely. This also explains to lower risk for relapse in our cohort as compared to previous studies [20]. Still, out of the GD 268 patients with blood samples (see Additional file 1: Figure S1) 25 and 26 received surgery or RAI in the long term, respectively and median time to definitive therapy after diagnosis was 35 months (median, IQR 8–71, mean 47 months) which argues against selection bias. Second, previous studies compared assay performance between GD patient and healthy volunteers, while we included patients with different types of thyroid pathologies. Thus, our results may better reflect real life indications for TRAb.

It is well known that TRAb levels decline gradually under ATD treatment until they disappear in about three quarters of the patients after 18 months [21]. In our opinion, this has a limited influence on our results as we only included patients up to 2.5 months after ATD initiation. By definition, every untreated GD patient should have TRAb. However, in the past up to 6–7% of GD patients were described to lack detectable TRAb, albeit these numbers are rather based on earlier TRAb assay generations [22, 23]. Nevertheless, in our study four sera of GD patients (4.8%) were negative with all assays.

Specificities ranged from 87.5% for the bioassay to 97.9% for the EliA anti-TSH-R at the upper cut-off (3.3 U/l). This is in agreement with the specificity of 97.7% published by Luther et al. [13] for the EliA anti-TSH-R. With EliA anti-TSH-R only one patient of the control group (autoimmune thyroiditis) had a borderline result (MOC 1.03 at cut-off 3.3). This serum was positive with all other assays (MOCs: RSR Fast TRAb 3.1, IMMULITE TSI 4.36, BRAHMS TRAK 1.39, TSAb Bioassay 1.29). Previously published specificities are generally higher (98.7–100%) compared to our results [8, 10, 11, 17, 19]. However, many studies included healthy subjects, whereas our control group consisted solely of thyroid-related disease patients. The frequency of TRAb positivity for multinodular toxic goiter or primary autoimmune hypothyroidism has been shown to be about 10% with RSR 3rd generation TRAb ELISA [17] and 10% for Hashimoto’s thyroiditis (HT) with BRAHMS TRAK [12]. According to the literature, stimulating TRAb can be found in 5.5–22% of HT patients [24, 25]. TRAb were detected in 1 out of 15 patients (6.7%) in the HT-control group. This particular serum was positive with all binding assays (MOCs: RSR Fast TRAb 2.28, IMMULITE TSI 1.39, BRAHMS TRAK 1.08) except with EliA anti-TSH-R (MOC 0.79 at cut-off 2.9) and TSAb bioassay (MOC 0.57). In this case both TSAb and TBAb bioassays were negative. According to Diana et al. TBAb can be observed in 4.2% of GD and in 9.3% of HT patients [26]. In our study, TBAb were detected in low amount in only one patient with silent thyroiditis (data not shown). This could be due to the different bioassay setup used in the study by Diana et al. [26] or to the limited sample size of our retrospective analysis.

### Prediction of relapse

Added to the GREAT score two assays (i.e. BRAHMS TRAK, and IMMULITE TSI) showed a statistically significant improvement of its predictive capabilities. Thus, these assays might provide a clinical benefit in predicting the relapse risk of newly diagnosed GD patients offered ATD therapy.

Somewhat surprising was the finding that concentrations of EliA anti-TSH-R did not seem to differ largely between the two groups (see Tables 1 and 2 for medians and Fig. 3 for box-plots), whereas the average HR for relapse prediction for the assay itself was the highest of all (see column “HR for assay alone (Q4 vs. Q1-3) (95% CI)” in Table 4). We think that this finding occurred by chance due to our small sample size as suggested by the wide confidence intervals. In this subsample of our previously published dataset [6], we observed a rather low overall recurrence rate of only 21.7% (originally 50.1%). This is slightly lower than usually reported from other cohorts in the past (30–60%) [9–11]. Although we had such a low incidence of events, we still observed statistically significant findings. Thus, we are confident that our data are robust and valid. Especially, as we ensured a high follow up rate in our original study by performing follow-up interviews with patients and/or their primary care physicians in case there had not been a contact within the last 6 months with a study center. In Switzerland, patients typically stay with their general practitioner for many years.

The overall predictive accuracy of the TRAb assays alone is ranging from 0.67 to 0.71, being like the GREAT score with the routine TRAb (AUC of 0.69). Although some new TRAb assays showed statistically significant improvements, it is less clear if these improvements prove clinically relevant.

Fitted into a survival model, we compared the fourth quartile of TRAb assay results against the remaining lower three. HR for all TRAb assays were in the same range as those for the GREAT class II (i.e. HR 1.79; 95% CI 1.42–2.27). When added to the GREAT score predictive ability improved even further. Hence, we believe that the TRAb assays used in our study provide some benefit for patient assessment with only slight differences between the different manufacturers. There is a slight reduction in hazard ratios in GREAT class III, which we attribute mainly to the variance caused by few data points in this group.

All these findings do not apply to the cAMP bioassay. Although disease course prediction has been reported to be improved by using bioassays, we could not replicate similar results [27, 28]. Even the IMMULITE TSI assay by Siemens did not have unrivalled predictive capabilities, albeit it is supposed to specifically detect only stimulatory antibodies. One reason might be that our sample size has not been large enough for a confirmatory finding.

Overall, the fact that a single factor in predicting the outcome of GD patients under ATD therapy is insufficient and needs to be combined with other factors. Accordingly, the addition of the new assays to the GREAT score is better than the predictive power of the assays alone. This also explains why previous attempts to predict relapse risk have failed [4, 7, 11–17]. Additionally, it leaves ample space for further research, either on even more specific TRAb or entirely new biomarkers (e.g. cytokines, genetic markers).

We acknowledge several limitations in our study. First, this study is retrospective in design. However, we could gather most data from medical records and we have a long enough follow-up. Second, although we analyzed the blood samples of 332 patients, we had to exclude all but 83 from analysis because a lot of samples were drawn long after ATD treatment initiation. As an exclusion criterion, we chose an ongoing ATD therapy duration for more than 2.5 months. We randomly chose this cut-off as it allowed us to use approximately 1/3 of our dataset. Although, there is a steady fall in TRAb levels during ATD treatment, we do not think that this has inflicted our results. Whereas TRAb levels seem to fall more strongly within 1–3 months after thyroidectomy [29], this decline is less pronounced in patients receiving ATD therapy [30–32]. Thus, we think that including blood samples from patients being up to 2.5 months under ATD therapy did not introduce substantial bias.

Third, we have longer treatment times than recommended by current evidence [4, 33]. Median treatment time was similar in both groups (19 vs. 18 months). This is explained by our retrospective design. Physicians and patients usually opt for an extended medical therapy before referral to a thyroid ablative procedure. We hold it unlikely that this might have influenced the results, as treatment duration over 18 months have been found to be of no benefit regarding relapse rate [4].

Forth, our study centers used different routine TRAb assays over the time course of our study. One might argue, that this might have introduce bias. In this case, it should be expected that our results were shifted towards non-significant findings as it disperses our baseline values. Nevertheless, we still found good prognostic accuracy despite inconsistencies in our data set compared to the one from the original GREAT score publication [5], underscoring the consistency of the GREAT score.

Fifth, we used a convenience sample based on a biological repository and had only limited samples available for measurement of TRAbs. Also, we did not use the novel Thyretain bioassay which may have much better performance compared to older bioassays [34]. This should be evaluated in future studies.

Finally, due to our inclusion criteria, seronegative patients with Graves’ hyperthyroidism are not represented in our study and it remains unclear how well our findings apply to this patient population. However, every new TRAb assay generation into clinical practice has reduced this population further [19]. It is believed that even those seronegative have TRAb production confined to the thyroid itself or adjacent lymph nodes [35].

## Conclusions

Based on this retrospective analysis, all the studied TRAb assays, but not the bioassay, seem to have better diagnostic and predictive abilities. Thus, they improve assessment of diagnosis and relapse risk in GD, which influences initial treatment decisions. Due to the small sample size and retrospective design with possible selection bias, our data need prospective validation.

## Additional file

Additional file 1: Supplementary information on the assays tested**. Table S1.** Specifications of routine assays used. **Figure S1.** Patient inclusion diagram. **Figure S2.** ROC graphs of TRAb assays with routine assay as reference. **Figure S3.** Kaplan-Meier-Survival graphs of TRAb assays fourth versus first to third quartile. (DOCX 373 kb)

## Abbreviations

ATD: Antithyroid drugs
AUC: Area under the receiver operator curve
GD: Graves’ disease
GREAT: Graves’ Recurrent Events After Therapy
HR: Hazard ratio
IQR: Interquartile range
ROC: Anti-thyroperoxidase
T4 and T3: Thyroxine and triiodothyronine
TPO-Ab: Antibodies
TRAb: TSH-receptor antibody tests
TRAb: TSH-receptor autoantibodies
TSH: Thyrotropin
