# Supplementary material for: Comparison of Five TSH-Receptor Antibody Assays in Graves’ disease: results from an observational pilot study
Source: BMC Endocr Disord. 2019 Apr 25;19:38. doi: 10.1186/s12902-019-0363-6 (PMC6482584; doi:10.1186/s12902-019-0363-6)
Supplement: Supplementary file 1 — Supplementary information on the assays tested. Table S1. Specifications of routine assays used. Figure S1. Patient inclusion diagram. Figure S2. ROC graphs of TRAb assays with routine assay as reference. Figure S3. Kaplan-Meier-Survival graphs of TRAb assays fourth versus first to third quartile. (DOCX 373 kb) [file 12902_2019_363_MOESM1_ESM.docx]

**Comparison of Five TSH-Receptor Antibody Assays in Graves’ disease**

***Supplementary material***

*Tristan Struja^1^, ^*^Rebecca Jutzi^1^, ^*^Noemi Imahorn^1^, Marina Kaeslin^1^, Fabienne Boesiger^1^, Alexander Kutz^1^, Esther Mundwiler^2^, Andreas Huber^2^, Marius Kraenzlin^3,4^, Beat Mueller^1,3^, Christian Meier^3,4^, ^†^Luca Bernasconi^2^ and ^†^Philipp Schuetz^1,3^

*equally contributing first authors

^†^equally contributing senior authors

[tristan.struja@gmail.com](mailto:tristan.struja@gmail.com); rebecca.jutzi@stud.unibas.ch; noemi.imahorn@stud.unibas.ch; m.kaeslin@stud.unibas.ch; fabienne.boesiger@gmx.ch; kutz.alexander@gmail.com; [esther.mundwiler@ksa.ch](mailto:esther.mundwiler@ksa.ch); andreas.huber@ksa.ch; marius.kraenzlin@unibas.ch; [christian.meier@unibas.ch](mailto:christian.meier@unibas.ch); [happy.mueller@unibas.ch](mailto:happy.mueller@unibas.ch); [luca.bernasconi@ksa.ch](mailto:luca.bernasconi@ksa.ch); [schuetzph@gmail.com](mailto:schuetzph@gmail.com)

^1^ Medical University Department, Clinic for Endocrinology, Diabetes & Metabolism, Kantonsspital Aarau, Aarau, Switzerland; ^2^ Department of Laboratory Medicine, Kantonsspital Aarau, Switzerland; ^3^ Medical Faculty of the University of Basel, Switzerland; ^4^ Endonet, private practice, Basel, Switzerland

**Corresponding author and person to whom reprint requests should be addressed:**

Tristan Struja, MD

University Clinic of Medicine, Department of Endocrinology, Diabetes, and Metabolism, Kantonsspital Aarau

Tellstrasse, CH-5001 Aarau, Switzerland

tristan.struja@gmail.com (E-Mail)

**Funding and disclosure statement:** This study was supported in part by the Swiss National Science Foundation (SNSF Professorship, PP00P3_150531 / 1) and the Research Council of the Kantonsspital Aarau (1410.000.044). Funders had no role in the design, analysis or writing of this article. Commercial companies provided the testing reagents only (TSI Assay, Siemens; EliA anti-TSH-R, Thermo Fisher; TRAK human KRYPTOR, B·R·A·H·M·S Thermo Fisher; TRAb Fast ELISA, RSR). TSH receptor stimulating/blocking activities were assessed by bioassays at RSR facility (Cardiff, UK). The Sponsors do have no influence on study planning or publication. BM and PS received research support by ThermoFisher Scientific, Roche Diagnostics, Abbott and Siemens unrelated to this study. All authors confirm that they do not have a conflict of interest associated with this manuscript.

**Supplementary information on the assays tested**

BRAHMS TRAK human KRYPTOR (Thermo Fisher Scientific, Germany)

This assay is a fully automated immunoassay that uses a M22 TSH autoantibody coupled to a fluorescent acceptor. It is bound to the N-terminal part of a TSH-receptor. After incubation with the test serum and consecutive washing, a recombinant human TSH-receptor is added which is coupled with a fluorescent donator competing with the TRAb. Thus, signal intensity is inversely proportional to the TRAb bound. The assay is calibrated to WHO first international standard for TRAb, NIBC 90/672 and the reportable range is 0.27 – 20 U/L (cut-off 1.8 U/L). Intra-assay variance for the range of 1.2 to 2.0 U/L is reported as < 7.0%. Inter-assay variance for the range of 1.0 to 2.0 U/L is reported as < 18.0%.

IMMULITE 2000 TSI (Siemens, Healthineers, Germany),

This assay is an automated, two-cycle, chemiluminescent immunoassay. It uses a pair of recombinant human TSH-receptor constructs in a bridging immunoassay format. The capture receptor is coated onto a solid phase. The signal receptor is a recombinant human TSH-receptor. First, sample is incubated with the solid phase allowing the TSI to bind through one arm to the capture receptor. After washing out residual samples the signal receptor is added. By forming a bridge, the complexed TSI bind the signal receptor through the second arm. Unbound material is wash out again before chemiluminescent substrate is added and a signal is generated in direct relation to the amount of TSI. Assay is traceable to WHO second international standard for TRAb, NIBC 08/204 and the reportable range is 0.10 – 40 U/L (cut-off 0.55 U/L). Intra-assay and inter-assay variance at 0.69 U/L is reported 4.1% and 5.1%, respectively.

EliA anti-TSH-R (Thermo Fisher Scientific, Germany)

The whole test is automatically processed on the Phadia 250 platform. It is a competitive enzyme immunoassay. The wells are coated with recombinant human TSH-receptors. TRAb of the probe will bind to the coating and after washing of unbound components, the enzyme-tagged recombinant EliA anti-TSH-receptor conjugate is being added. After incubation and another wash-out, a development solution is added and lastly the activity of the fluorescence is measured. The less fluorescence the less conjugate is bound to the coating. Hence, the probe contains more TRAb. The system is calibrated according to the second international standard NIBSC 08/204. The lower and upper limit of detection are 1.5 U/L and 80 U/L, respectively. Intra-assay and inter-assay variance at 3.2 U/L (positive cut-off >3.3 U/L, negative cut-off <2.9 U/L) is reported 10.6% and 11.4%, respectively.

ELISA RSR TRAb Fast (RSR Limited, UK).

TRAb in patients’ sera are allowed to interact with TSH-receptor coated onto ELISA plate wells. After washing out residue, a recombinant human M22-peroxidase TSH autoantibody is, where it interacts with immobilized TSH-receptor not already blocked by bound TRAb. The amount of M22-Peroxidase bound to the plate is then determined by the addition of fluorescence solution. A lower absorbance indicates the presence of TRAb in the test sample. The assay is calibrated according to NIBSC 90/672 and the measuring range is 1 – 40 U/L (positive cut-off ≥1.0 U/L, lower detection limit at 2 SD 0.16 U/L). Intra-assay and inter-assay variance at 2.0 and 4.6 U/L is reported 7.2% and 3.3%, respectively

BioassayRSR™ TSAb and TSBAb

CHO cells stably transfected with the wild type human TSH-receptors are seeded into 96 well plates at 25000 cells/well and incubated at 37°C/5% CO_2_ for 48 hours. For the detection of stimulating type autoantibodies patient sera is diluted 1/10 in cyclic AMP assay buffer. For detection of blocking TSHR autoantibodies patient sera is diluted in cyclic AMP assay buffer containing TSH to give a final 1/10 dilution of serum (3ng/mL of TSH in the cell well). Healthy blood donor serum diluted 1/10 in cyclic AMP assay buffer is used as a negative control. Healthy blood donor serum diluted in cyclic AMP assay buffer containing TSH to give a final 1/10 dilution of serum is used as a positive control. The diluted serum samples are incubated in the cell wells for 1 hour at 37°C/5% CO_2_. The test samples are removed and the cells are lysed using HCl to release the intracellular cAMP. The lysates are assayed for cAMP using the Direct Cyclic AMP ELISA kit from Enzo Life Sciences. Results are expressed as pmol/mL. Stimulating activity is the increase in intracellular cAMP production via activation of the TSHR. A positive result is ≥ 150% stimulation compared to the healthy blood donor control. The equation for calculating the % stimulation is: 100 x (pmol/mL of cAMP in test sample / pmol/mL of cAMP in healthy blood donor). Blocking activity is the inhibition of TSH induced stimulation of cyclic AMP production. A positive result ≥ 30% inhibition of TSH stimulation in healthy blood donor serum. The equation for calculating the blocking activity is: 100 x (1-[ pmol/mL of cAMP in test sample +TSH / pmol/mL of cAMP in healthy blood donor +TSH]).

**Supplementary table 1 Specifications of routine assays used**

|  | **Kantonsspital Aarau** | **Endonet Outpatient Clinic** |
| --- | --- | --- |
| **Timeframe 2010-2014** | | |
| **TSH** | After 01/2011 Dimension Vista, Siemens, Germany  Up to 12/2010 Immulite-TSH, Siemens, Germany | Elecsys TSH Test, Cobas e411, Roche, Switzerland  Coefficient of variation 1.5-8.6%  Lower limit of detection 0.014 U/l |
| **fT4** | After 01/2011 Dimension Vista fT4, Siemens, Germany  Up to 12/2010 Immulite-fT4, Siemens, Germany | Elecsys fT4 Test, Cobas e411, Roche, Switzerland  Coefficient of variation 1.3-4.0%  Lower limit of detection 0.3 pmol/l |
| **fT3** | After 01/2011 Dimension Vista, Siemens, Germany  Up to 12/2010 Immulite-fT3, Siemens, Germany | N/A |
| **T3** | Immulite-T3, Siemens, Germany | Elecsys T3 Test, Cobas e411, Roche, Switzerland  Coefficient of variation 3.6-5.3%  Lower limit of detection 0.3 nmol/l |
| **TPO-Ab** | ELISA, INOVA Diagnostics, USA | Elecsys TPO-Ab Test, Cobas e411, Roche, Switzerland  Coefficient of variation 4.2-7.0%  Lower limit of detection <5.0 U/l |
| **TRAb** | RSR ELISA (3^rd^ generation), RSR Ltd., UK  Cut-Off < 0.4 U/l  Lower limit of detection 0.4U/l | BRAHMS TRAK human LIA, ThermoScientific, Germany  Coefficient of variation 20% 0.8-1.2U/l  Cut- off < 0.4U/l  Lower limit of detection 0.4U/l |
| **Timeframe 2006-2010** | | |
| **TSH** | Immulite-TSH, Siemens, Germany | Elecsys 2010, Roche, Switzerland  Coefficient of variation 1.5-8.6%  Lower limit of detection 0.014 U/l |
| **fT4** | Immulite-fT4 Siemens, Germany | Elecsys 2010, Roche, Switzerland  Coefficient of variation 1.3-4.0%  Lower limit of detection 0.3 pmol/l |
| **fT3** | Immulite-fT3, Siemens, Germany | N/A |
| **T3** | Immulite-T3, Siemens, Germany | Elecsys 2010, Roche, Switzerland  Coefficient of variation 3.6-5.3%  Lower limit of detection 0.3 nmol/l |
| **TPO-Ab** | ELISA, INOVA Diagnostics, USA | Elecsys 2010, Roche, Switzerland  Coefficient of variation 4.2-7.0%  Lower limit of detection <5.0 U/l |
| **TRAb** | RSR ELISA (2^nd^ generation), RSR Ltd., UK  Cut-Off < 1.8 U/l  Lower limit of detection 0.4U/L | BRAHMS TRAK human LIA, ThermoScientific, Germany  Coefficient of variation 20% 0.8-1.2U/l  Cut- off < 0.4U/l  Lower limit of detection 0.4U/l |
| **Timeframe 2004-2006** | | |
| **TSH** | Immulite-TSH, Siemens, Germany | Elecsys 2010, Roche, Switzerland  Coefficient of variation 1.5-8.6%  Lower limit of detection 0.014 U/l |
| **fT4** | Immulite-fT4 Siemens, Germany | Elecsys 2010, Roche, Switzerland  Coefficient of variation 1.3-4.0%  Lower limit of detection < 0.3 pmol/l |
| **fT3** | Immulite-fT3, Siemens, Germany | N/A |
| **T3** | Immulite-T3, Siemens, Germany | Elecsys 2010, Roche, Switzerland  Coefficient of variation 3.6-5.3%  Lower limit of detection 0.3 nmol/l |
| **TPO-Ab** | ELISA, INOVA Diagnostics, USA | Elecsys 2010, Roche, Switzerland  Coefficient of variation 4.2-7.0%  Lower limit of detection <5.0 U/l |
| **TRAb** | RSR ELISA (2^nd^ generation), RSR Ltd., UK  Cut-Off < 1.8 U/l  Lower limit of detection 0.4U/L | BRAHMS TRAK human LIA, ThermoScientific, Germany  Coefficient of variation 20% 0.8-1.2U/l  Cut- off < 0.4U/l  Lower limit of detection < 0.4U/l |

**Supplementary figure 1 Patient inclusion diagram**

**
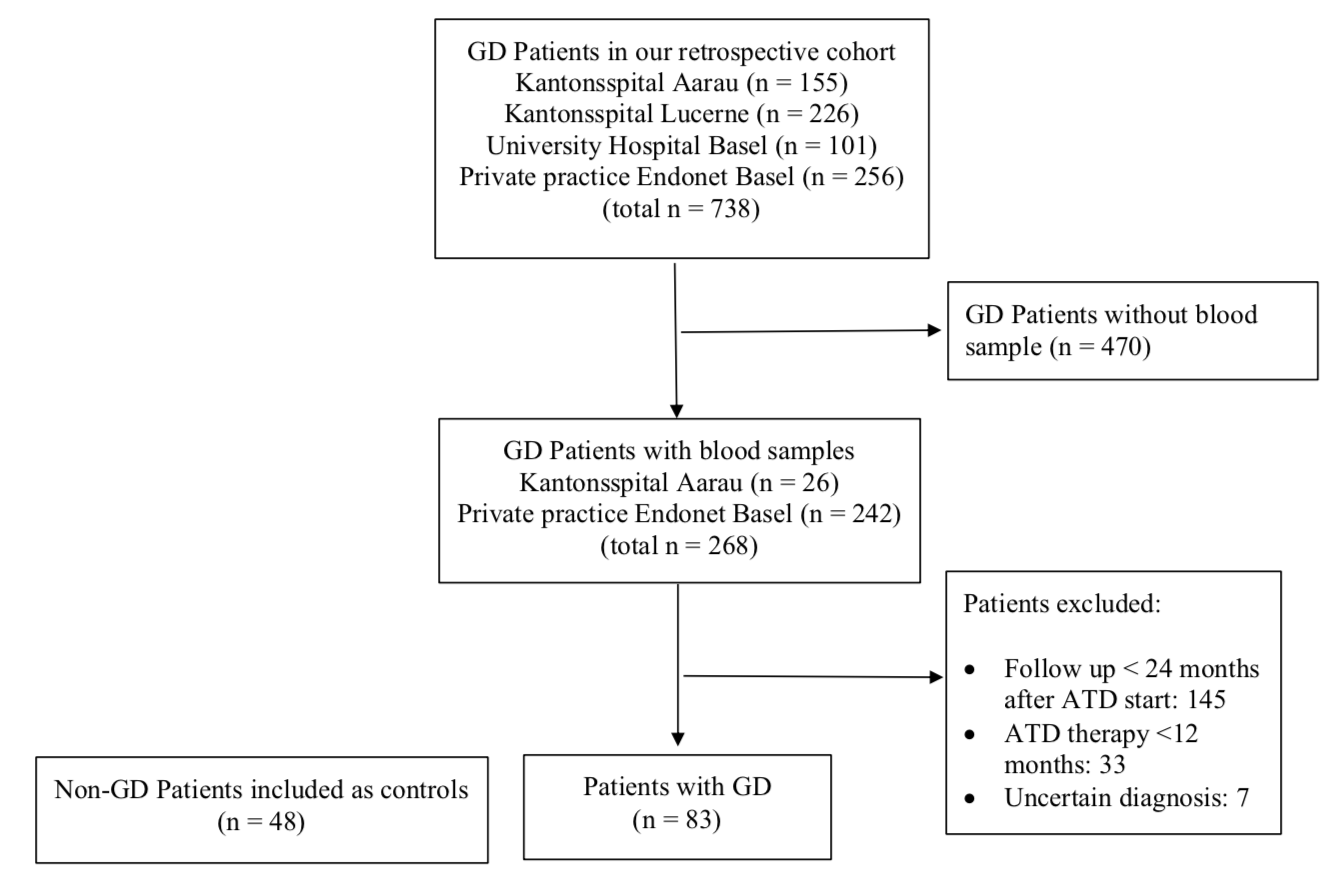
**

**Supplementary figure 2 ROC graphs of TRAb assays with routine assay as reference**

**Supplementary figure 3 Kaplan-Meier-Survival graphs of TRAb assays fourth versus first to third quartile**
